# Ki-67 Expression as a Prognostic Marker: A Comparative Immunohistochemical Analysis of Oral Epithelial Dysplasia and Oral Squamous Cell Carcinoma

**DOI:** 10.7759/cureus.38941

**Published:** 2023-05-12

**Authors:** Vineet Gupta, Karthikeyan Ramalingam, Dinesh Yasothkumar, Diptakshi Debnath, Vinay Sundar

**Affiliations:** 1 Oral Pathology, Maharaj Ganga Singh Dental College and Research Centre, Sri Ganganagar, IND; 2 Oral Pathology and Microbiology, Saveetha Dental College and Hospitals, Chennai, IND; 3 Oral Pathology and Microbiology, Saveetha Institute of Medical and Technical Sciences, Chennai, IND; 4 Oral and Maxillofacial Surgery, Saveetha Dental College and Hospitals, Chennai, IND; 5 Oral and Maxillofacial Surgery, Saveetha Institute of Medical and Technical Sciences, Chennai, IND

**Keywords:** ki67, grading, proliferation marker, immunohistochemistry, prognosis, oral squamous cell carcinoma, oral epithelial dysplasia, prognostic marker, ki 67

## Abstract

Introduction

Oral dysplasia is a frequent precancerous condition that may lead to oral cancer. The histopathologic abnormalities exhibited in a chronic, progressive, and premalignant condition of the oral mucosa are referred to as oral epithelial dysplasia (OED). It might show up as erythroplakia, leukoplakia, or leukoerythroplakia. OED is a premalignancy histologic marker that predicts a higher likelihood of squamous cell carcinoma development.

Aims and objectives

The aim of this study is to identify an association between Ki-67 protein expression and histological grading of OED and oral squamous cell carcinoma (OSCC) and to compare the expression of Ki-67 in different grades of OED and OSCC with the prognosis.

Materials and methods

The current retrospective research is focused on evaluating epithelial dysplasia and analyzing the function of Ki-67 as a prognostic marker after receiving institutional ethical approval. Group I - normal oral mucosa (NOM), Group II - OED, and Group III - OSCC were included in the study. For statistical analysis, SPSS Statistics version 21.0 (IBM Corp. Released 2021. IBM SPSS Statistics for Windows, Version 28.0. Armonk, NY: IBM Corp) was utilized. The Cox regression model was employed to look at interactions between various prognostic variables. At p<0.05, differences were deemed statistically significant.

Results

Ki-67 expression was confined to the basal layers in the normal oral epithelium and in the basal, suprabasal, and spinous layers in OED. Ki-67 positive cells were mostly found on the perimeter of well, moderate, and poorly differentiated OSCC tumor nests with Ki-67 positive cells scattered throughout OSCC. According to statistical analysis, there is a substantial difference in expression between OED and NOM, OSCC and NOM, and OED and OSCC.

Conclusion

Our study showed that there is a progressive increase in Ki-67 expression across various grades of OED, and the highest expression was noted in OSCC. Early identification and prompt treatment will help in improving the quality of life of such patients.

## Introduction

Oral dysplasia is a potentially malignant disorder that may lead to oral cancer, but the chance of developing oral cancer varies greatly, ranging from 6% to 36% [1]. The histopathologic abnormalities exhibited as a chronic and progressive potentially malignant disorder of the oral mucosa are referred to as oral epithelial dysplasia (OED). It might present as erythroplakia, leukoplakia, or leukoerythroplakia. Some individuals can present even with early invasive squamous cell carcinoma and dysplastic changes in adjacent normal mucosa [2].

According to a recent WHO report, there is no apparent agreement on the best grading system for OED that is also clinically relevant [3]. The accuracy of grading is determined by the quality of tissue procured and the location of the biopsy. The grading of dysplasia is also subjective, with a wide inter-observer and intra-observer variation [4,5]. Cellular atypia, lack of normal development, and stratification are dysplastic hallmarks of the stratified squamous epithelium [6]. It has been shown that the accumulation of genetic and epigenetic abnormalities occurs throughout the malignant transformation and can be visualized in the oral tissues as OED [7,8].

Previous research has shown that elevated Ki-67 indices are associated with disease progression and a poor prognosis in oral squamous cell carcinoma (OSCC) [9]. In OSCC, cell proliferation as evaluated by Ki-67 expression at the invasive tumor front has been shown to have a substantial positive connection with histologic grading of the malignancy [10]. Hence, our study was performed to assess the expression of Ki-67 in the normal oral mucosa (NOM), OED, and OSCC along with its clinical correlation as a prognostic marker.

## Materials and methods

This retrospective immunohistochemical study was followed in accordance with the ethical standards of the Institutional Human Ethical Committee on human experimentation after receiving approval vide IHEC/SDC/PhD/OPATH-2212/22/001 and was in accordance with the Helsinki Declaration of 1975, as revised in 2000. The formula used for calculating the adequate sample size in our study is n=Z2P(1−P)/d2, where n is the sample size, Z is the statistic corresponding to the level of confidence, P is the expected prevalence (that can be obtained from the same study or a pilot study conducted by the researchers), and d is precision (corresponding to effect size). The level of confidence was 95%, and the required sample count was 30.

Hence, this retrospective study was performed using 35 samples, and its biopsy request details were procured from the department's archives. The samples consisted of 30 formalin-fixed paraffin-embedded tissue blocks of OED (five mild OED, five moderate OED, and five severe OED) and OSCC (five well-differentiated OSCC, five moderately differentiated OSCC, and five poorly differentiated OSCC) along with five tissue blocks of the NOM as controls. Inclusion criteria were cases with primary OSCC, oral leukoplakia, and oral erythroplakia. Exclusion criteria were recurrent cases of OSCC, other malignancies of the head and neck region, and patients with tumor-related immunodeficiency syndromes.

Clinical information about the patients was retrieved and analyzed. The demographic variables include gender, habits, and age. The pathologic variables include the pattern of invasion, the grade of the tumor, lymphovascular invasion, the depth of invasion, stromal type, and neural invasion. The clinical variables include tumor size, site, distant metastasis, local metastasis, hemoglobin levels, and clinical stage. Patient outcomes include recurrence, disease-free survival, and death if any were noted.

3µ paraffin sections of formalin-fixed tissues were prepared using Leica RM2245 semi-automated microtome (Leica GMBH, Germany). The sections were used for both histological and immunohistochemical evaluation. H&E stained sections were used for routine histological examinations. The lesions were histopathologically classified using the criteria established by the WHO working group on oral cavity cancers.

The clinical and histological diagnoses were not disclosed to the examining oral pathologists. The findings were separately recorded on individual score sheets, and the observer assigned a final evaluation to each instance as “no dysplasia/mild/moderate/severe dysplasia.” The dysplastic features were graded as follows: mild dysplasia (the minimal requirements for dysplasia include architectural disruption confined to the bottom third of the epithelium, as well as cytological atypia), moderate dysplasia (the first criterion for identifying this group was the architectural disruption that extended into the middle part of the epithelium), and severe dysplasia (more than two-thirds of the epithelium exhibited structural disruption with cytologic atypia to be diagnosed as severe dysplasia). However, taking into account that irrespective of how widely the atypical cells covered the epithelium, lesions that demonstrated significant cytological changes were raised to a higher grade of dysplasia.

3µ tissue sections were also prepared on positively charged slides and subjected to Ki-67 immunohistochemical staining as per the manufacturer’s instructions (Biogenex, USA). Two independent oral pathologists assessed the immunostaining. To achieve a consensus, differences of opinion were evaluated collectively. Positive expression was defined as cells that stained brown color. The percentage of Ki-67-positive cells to total cells was calculated, and three distinct fields were investigated.

On a four-point scale from 0 to 3, this marker was rated in the epithelium based on the region of expression as follows: Score 0 (no expression), Score 1 (expression in the basal layer only), Score 2 (expression in basal and suprabasal layers only), and Score 3 (expression in basal, suprabasal, and spinous layers). The percentage of cells with positive immunoexpression was also calculated. In each segment, the region with the greatest number of positive cells was evaluated. The area of expression was rated as Score 0 (negative, no expression), Score 1 (if there were 1% to 25% positive epithelial cells), Score 2 (if there were 25% to 50% positive cells), and Score 3 (if there were more than 50% positive cells).

For statistical analysis, SPSS Statistics version 21.0 (IBM Corp. Released 2021. IBM SPSS Statistics for Windows, Version 28.0. Armonk, NY: IBM Corp.) was utilized. The Cox regression model was employed to look at interactions between various prognostic variables. At P<0.05, differences were deemed statistically significant.

## Results

**Figure 1:**
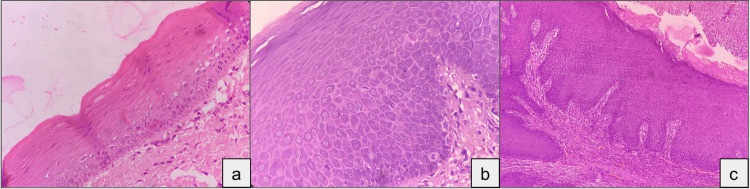
Photomicrographs showing epithelial dysplasia (H&E, 10x) Photomicrographs showing (a) mild epithelial dysplasia, (b) moderate epithelial dysplasia, and (c) severe epithelial dysplasia

**Figure 2:**
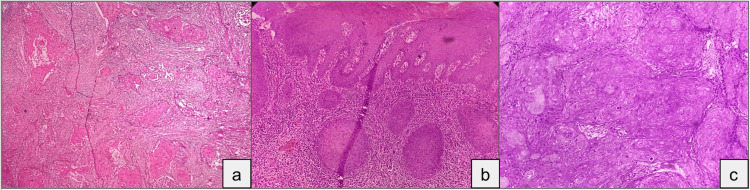
Photomicrographs of squamous cell carcinoma (H&E, 10x) Photomicrograph showing (a) well-differentiated squamous cell carcinoma, (b) moderately differentiated squamous cell carcinoma, and (c) poorly differentiated squamous cell carcinoma

The current retrospective research focused on evaluating epithelial dysplasia and analyzing the function of Ki-67 as a marker of OSCC. In the present study, the samples were studied among the three groups: NOM comprised five samples, and 15 samples of OED and OSCC were analyzed (Figures 1, 2).

Out of 30 samples studied, 60% were men and 40% were women. The mean age was 51 years for men and 44 years for women. About 85% of patients had tobacco usage in the form of smoking or chewing.

Out of 15 OSCC patients, seven OSCC presented in buccal mucosa and gingivobuccal sulcus, four presented in the tongue, three presented in the alveolus, and one in the floor of the mouth. Clinical staging showed that nine were in Stage IV disease, five were in Stage III disease, and one was in Stage II disease. Mean hemoglobin levels were 13.9 gm/dl in men and 12.2 gm/dl in women. All of our patients were under follow-up for two years now and did not show any recurrence or mortality during the study.

In our histopathological evaluation, 13 samples had a Type III pattern of invasion showing invasive tumor islands with >15 cells cluster. Two samples had a Type II pattern of invasion showing solid cords and strands. We did not find any other pattern of invasion among the studied samples. We did not find any lymphovascular invasion or neural invasion or distant metastasis.

**Figure 3:**
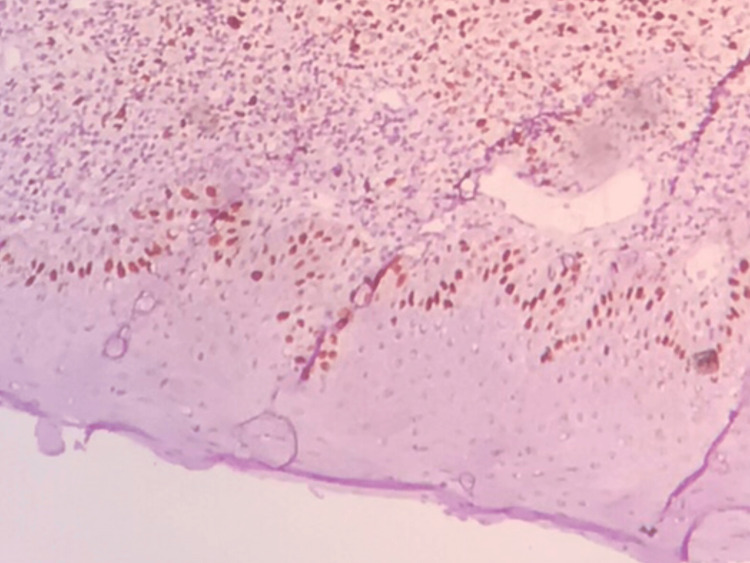
Photomicrograph showing Ki-67 expression in OED (IHC, 10x) Photomicrograph showing Ki-67-positive cells in the dysplastic epithelium

**Figure 4:**
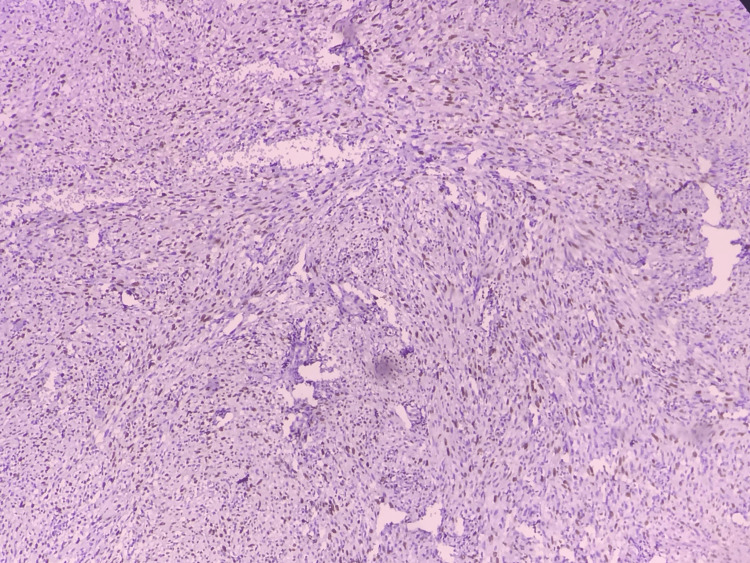
Photomicrograph showing Ki-67 expression in OSCC (IHC, 10x) Photomicrograph showing Ki-67 expression observed in OSCC group

Ki-67 expression was performed and analyzed in the study groups (Figures 3, 4). The presence of Ki-67 stained cells was found in the basal layer for Group (INOM), the presence of stained cells in basal, suprabasal, and spinous layers for Group II (OED), and in Group III (OSCC), all the epithelial layers showed Ki-67 positivity.

**Figure 5:**
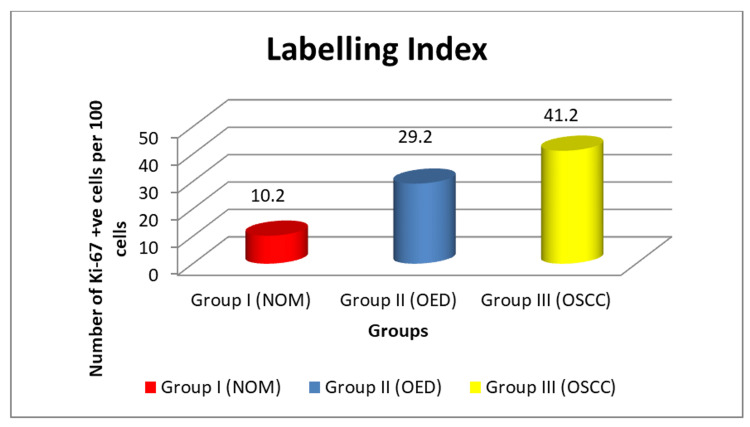
Graphical representation of Ki-67 positivity in the study Graph showing the mean number of cells stained with Ki-67 according to labeling index scores among different study groups: Group I (NOM), Group II (OED), and Group III (OSCC)

Immunohistochemical quantitative results revealed that the average mean number of Ki-67 positive cells was 10.2 in NOM, 29.2 in OED samples, and 41.2 in OSCC samples as observed (Figure 5).

**Figure 6:**
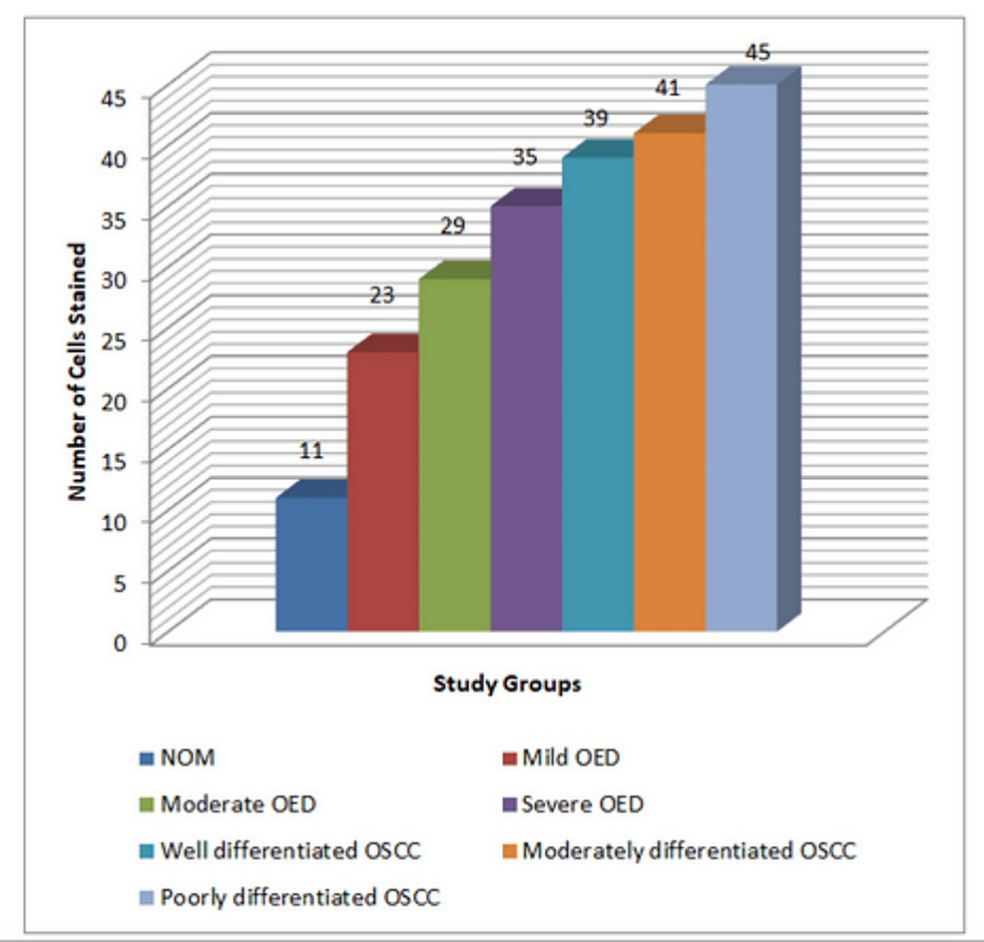
Graph showing the quantitative Ki-67 expression among the studied samples Graphical demonstration of immunohistochemical quantitative results of the Ki-67 protein expression in study samples: Group I (NOM), Group II (OED), and Group III (OSCC)

On average (Figure 6), the number of cells stained with Ki-67 was found to be 11 in NOM, 23 in mild OED, 29 in moderate OED, and 35 in severe OED samples. The mean number of Ki-67 stained cells was 39 in WDSCC, 41 in MDSCC, and 45 in poorly differentiated SCC samples.

Only peripheral expression of Ki-67 was noted in tumor nests of well-differentiated OSCC. The periphery and center of tumor nests of moderately differentiated OSCC showed Ki-67 expression. Diffuse expression of Ki-67 along with expression in the periphery and center of tumor nests were noted in poorly differentiated OSCC (Table 1).

**Table 1:** Table summarizing the immunohistochemical expression of Ki-67 among the study samples Table showing the immunohistochemically stained cells in different layers among various study groups: Group I (NOM), Group II (OED), and Group III (OSCC) OED: oral epithelial dysplasia, OSCC: oral squamous cell carcinoma

Ki-67 expression among various study groups					
	Basal layer	Suprabasal and spinous layer	Tumour nests (periphery)	Tumour nests (periphery and centre)	Diffuse
NOM	Present	-	-	-	-
OED: mild	Present	Present	-	-	-
OED: moderate	Present	Present	-	-	-
OED: severe	Present	Present	-	-	-
OSCC: well differentiated	Present	Present	Present	-	-
OSCC: moderately differentiated	Present	Present	Present	Present	-
OSCC: poorly differentiated	Present	Present	Present	Present	Present

Table 2 evaluated groups II and III, the mean difference was found to be 12.01±0.31, and a statistically significant result was observed (p-value = 0.001).

**Table 2:** Comparison of study groups II and III with respect to Ki-67 expression Table showing the comparison of Ki-67 expression among OED and oral OSCC OED: oral epithelial dysplasia, OSCC: oral squamous cell carcinoma

	N	Mean difference±SD	Mean	SD	t-value	p-value
Group II: OED	15	12.01±0.31	29.2	0.41	0.43	0.001*
Group III: OSCC	15		41.2	0.51		
Test applied: Student’s t-test, *p <0.05 significant						

Similarly, Table 3 compared Group I and Group III which depicted that the mean difference of stained cells values (31.1±0.44) was statistically significant (p-value = 0.05).

**Table 3:** Table comparing the Ki-67 expression among groups I and III Table showing the comparison of NOM and OSCC samples with respect to Ki-67 expression NOM: normal oral mucosa, OSCC: oral squamous cell carcinoma

	N	Mean difference±SD	Mean	SD	t-value	p-value
Group I: NOM	5	31.1±0.44	10.2	0.32	0.33	0.05*
Group III: OSCC	15		41.2	0.51		

The mean comparison of the three study groups was found to be statistically significant (<0.001) (Table 4).

**Table 4:** Table comparing Ki-67 expression in the study Table showing the comparison of Ki-67 expression among various study groups NOM: normal oral mucosa, OED: oral epithelial dysplasia, OSCC: oral squamous cell carcinoma

	N	Mean	SD	p-value
Group I: NOM	5	10.2	0.32	<0.001*
Group II: OED	15	29.2	0.41	
Group III: OSCC	15	41.2	0.51	

Kappa statistics between the two observers also revealed % of agreement 94.5945945945946%, Cohen’s k 0.4714285714285713, and moderate agreement.

## Discussion

The minimum requirements for mild dysplasia include architectural disruption restricted to the bottom third of the epithelium, followed by modest cytological atypia. The first requirement for distinguishing moderate dysplasia is the architectural disruption that extends into the middle part of the epithelium. More than two-thirds of the epithelium must display architectural disruption with cytological atypia to be diagnosed as severe dysplasia. Different grades of OED have been discussed by many authors in the literature [2,10-14].

The Ki-67 protein has been widely studied as a molecular marker of proliferating cells in OED and OSCC, and the number of proliferating cells increases with dysplasia grade. All three phases of the cell division cycle (G1, S, and G2), as well as mitosis, depict the presence of Ki-67, but it is absent in quiescent or resting cells in the G0 phase [15-22]. In the final stages of mitosis, Ki-67 levels drop dramatically. Ki-67 has been found to play a crucial role in carcinogenesis, serving as a marker of tumor aggressiveness [23-27]. Furthermore, Ki-67 has also been implicated in tumor proliferation and invasion along with other markers like Bcl2 [28,29].

The biological activity of neoplasms is influenced by cell proliferation indicators. Ki-67 is thought to be one of the strongest indicators of survival and recurrence. Increased cell proliferation characterizes the shift of the normal oral epithelium to dysplasia and cancer. Thus, the Ki-67 protein may be used as a prognostic indicator for malignant transformation [26-29]. Hence, immunohistochemical expression of the cell cycle-associated protein Ki-67 was assessed in OED, OSCC, and NOM as a control in this study.

Ki-67 expression as measured by immunostaining has become the gold standard, with a threshold level of 10-14% positively stained cells indicating a high risk [23]. Furthermore, the relevance of the Ki-67 protein as an indicator in the diagnostic and prognosis evaluation of the severity of OED and histological grades of OSCC is discussed in this study. The mean labeling index score for NOM, OED, and OSCC was 10.2±0.32, 29.2±0.41, and 41.2±0.51, respectively. The growing cells in the normal oral epithelium were mostly found in the basal layer, which was consistent with earlier research [24,25].

The expression of the Ki-67 protein was observed in the basal, parabasal, and spinous layers of the epithelium of the OED in our investigation, and its expression increased with the degree of dysplasia. This was in line with earlier research [23,24]. The rate of malignant transformation may be influenced by the degree of dysplasia, with high-grade dysplastic lesions being 4.5 times more likely to undergo such transformation than low-grade dysplastic lesions [23-26].

In mild OED, the maximum expression of Ki‑67 was located at the basal and parabasal layers of the epithelium. There was no statistically significant difference between the mild OED and the NOM in our study. Hence, it may be inferred that predicting the prognosis of mild OED lesions is very difficult as their proliferative activity is comparable to normal epithelium.

Nuclear Ki-67 positivity was observed in the basal, parabasal, and some spinous layers of the epithelium in the moderate OED. There was a statistically significant difference between the moderate OED and the NOM, and nuclear Ki-67 positivity was found in the basal, parabasal, and most of the spinous layers of the epithelium in the severe OED. Hence, lesions with moderate to severe dysplasia have a higher risk of malignant transformation and should be monitored closely.

It was observed that the Ki-67 protein increased with advancing grades of OSCC which was also consistent with earlier research [23-28]. Nuclear Ki-67 positivity was identified in the peripheral region of tumor islands in well-differentiated OSCC. This suggests that the peripheral layer of the tumor islands contains less differentiated cells, but the core region contains highly differentiated cells capable of keratinization. As a result, no Ki-67 expression was detected in the tumor island's center cells.

Nuclear Ki-67 positivity was identified in the periphery region and part of the core area of tumor islands in moderately differentiated OSCC. Ki-67 staining was more quantifiable in moderately differentiated OSCC than in well-differentiated OSCC. Nuclear Ki-67 positivity was identified in the periphery region, and most of the center area of tumor islands had diffuse Ki-67 staining of poorly differentiated OSCC. Hence, it can be assumed that the cells were less differentiated and in a more proliferative phase.

We have observed that Ki-67 protein levels rise with the de-differentiation of OED and OSCC. The increased expression of the Ki-67 protein in OSCC might play a key role in the disease progression. This signifies that dysplastic epithelial cells have a high risk of malignant transformation. Hence, the Ki-67 protein is a potential therapeutic target in cancer due to its expression in all proliferating cells and the prognostic value of the Ki-67 marker in many cancers. Strategies that inactivate Ki-67 protein are promising for the future [29]. Finally, the growth rate of cells in OED and OSCC may be measured using Ki-67 as a prognostic and predictive marker.

Limitations

One limitation is the sample size, as only five individual variants of OED and OSCC were analyzed in our study. Further studies with larger sample sizes of individual entities will add more valuable information about the pathogenesis of OED and OSCC in correlation with proliferation markers like Ki-67. Longitudinal follow-up studies to monitor the progression with continuous clinical and histopathological analysis will throw light into this unexplored territory.

## Conclusions

Many studies have revealed that increased cell multiplication is observed in the transition from normal mucosa to epithelial dysplasia to frank malignancy. Hence, the rate of cellular proliferation can be assessed in correlation with the severity of OED and the grading of OSCC. Our findings of consistent and progressive increase in Ki-67 expression from NOM to OED to OSCC. Ki-67 expression increased with advanced dysplasia and higher grades of malignancy. Mild dysplasia had comparable Ki-67 expression to the NOM. Our study supports the fact that Ki-67 might be employed as a prognostic biomarker for the histological grading of OED and OSCC to establish the prognosis of such patients. A larger sample size of various grades of OED and subtypes of OSCC will enhance the knowledge about proliferation. Prompt identification of such proliferation markers can identify hyperactive lesions, and appropriate treatment can be initiated to avoid morbidity and fatal complications.
